# Annualized hospitalization rate with natalizumab vs fingolimod in second-line treatment for RRMS in the public healthcare system in Brazil: A claim database approach

**DOI:** 10.1371/journal.pone.0229768

**Published:** 2020-03-02

**Authors:** Guilherme Silva Julian, Ricardo Papaléo Rosim, Estela Cristina Carneseca, Jéssica Rigolon

**Affiliations:** 1 IQVIA Brasil, São Paulo—SP, Brazil; 2 Biogen Brasil, São Paulo—SP, Brazil; 3 Proestat Consultoria Estatística, Ribeirão Preto–SP, Brazil; Universita degli Studi di Napoli Federico II, ITALY

## Abstract

**Purpose:**

In the Brazilian public healthcare system, natalizumab is recommended as fourth-line treatment for relapsing-remitting multiple sclerosis (RRMS). Although natalizumab has already demonstrated higher effectiveness compared with fingolimod in some studies, this real-world study was conducted to evaluate annualized hospitalization rates (AHR) in Brazil for both treatments when switching from platform therapies. As secondary goals, we analyzed RRMS treatment patterns and hospitalization profiles.

**Material and methods:**

We extracted data from the DATASUS database of patients with MS (ICD-10 G35) who initiated treatment from January 2012 to December 2017. Two cohorts were screened for different purposes. Cohort 1 was used to analyze treatment patterns and hospitalization profiles and was defined as individuals who had at least one claim related to MS therapies and had received at least two lines of treatment. The second cohort, which was a subset of the first, was used to compare natalizumab’s and fingolimod’s AHR reduction from previous treatment lines and included patients switching from platform therapy to one of these two drugs. Cohort 2 adjustment was assessed through two different statistical methods: propensity score (PS) and inverse probability weighting (IPW).

**Results:**

Of 29,410 patients screened, 2,876 were included in cohort 1. Three quarters of hospitalizations reported in this cohort were for treatment of MS relapse. Cohort 2 included 1,005 patients, and natalizumab was more commonly used (n = 540) than fingolimod (n = 465). Both PS and IPW analyses showed that patients treated with natalizumab had a statistical significantly reduction in AHR compared with first-line treatment (p<0.01 for both PS and IPW), while fingolimod did not result in significant reduction in AHR (p = 0.20 for PS and p = 0.17 for IPW).

**Conclusion:**

This study provides real-world evidence of natalizumab’s and fingolimod’s effectiveness in terms of AHR, with an increased reduction in AHR with natalizumab. The findings of this study also provide information to support disease management and healthcare planning in the Brazilian public healthcare system.

## Introduction

Multiple sclerosis (MS) is a progressive inflammatory disease of the central nervous system that poses a disease burden to more than 2 million people worldwide [1]. The most common clinical course is relapsing-remitting multiple sclerosis (RRMS), accounting for 85% of all cases [2]. In Brazil, there is a high heterogeneity in prevalence rate among regions, with an estimated prevalence of 15 per 100,000 inhabitants (IC 95%: 6.0–12.6) [3]. The Northeast region of the country has the lowest reported prevalence rates (as low as 1.36 per 100,000 inhabitants), while the South region presents the highest reported MS prevalence (27.2 per 100,000 inhabitants) [4].

Currently, according to Brazilian Ministry of Health guidelines [3], the standard recommendation for first-line treatment of RRMS consists of interferon β, glatiramer acetate and teriflunomide. For second-line treatment, patients who were intolerant, nonadherent, or who experienced adverse reactions to those drugs should switch to another drug among the first-line treatment options or to dimethyl-fumarate (DMF). Patients with therapeutic failure or suboptimal response can switch not only to another drug among the first-line treatment options or DMF but also to fingolimod. The guidelines prioritize fingolimod for third-line treatment in cases for which it was not used in the second-line treatment. Finally, the use of natalizumab is indicated only for patients with therapeutic failure or a contraindication to fingolimod.

Although the Brazilian Ministry of Health recommends natalizumab for later lines of treatment, guidelines in other countries, such as the United States of America (US) and European Union, recommend natalizumab as second-line therapy [5,6]. In the US, natalizumab is suggested even as a first-line therapy [7]. In this context, real-world studies, particularly of treatment patterns and evaluations of effectiveness, play an essential role in guiding decision-making processes due to their external validity [8]. Natalizumab has demonstrated higher effectiveness when compared with other second- and third-line treatments, such as fingolimod [9–12].

In Brazil, local effectiveness data for RRMS drugs, including hospitalization rates and inpatient healthcare resource utilization, are limited. The present study primarily aims to evaluate and compare annualized hospitalization rates (AHR) for fingolimod and natalizumab after failure of platform treatments (interferons and glatiramer acetate). Additionally, we describe treatment patterns and inpatient healthcare resource utilization for patients with MS in Brazil. Such results may contribute to a better understanding of the healthcare resource utilization by patients with MS as well as natalizumab’s real-world effectiveness in Brazil and thus support decision-makers in the development of policies for improved care of patients with MS.

## Materials and methods

This study was performed in December 2018 with an analysis of outpatient and inpatient data for patients with MS (ICD-10 G35) from DATASUS claims databases from January 1, 2013, to December 31, 2017.

DATASUS is held by the Brazilian Ministry of Health Department of Informatics and provides data from procedures performed within the Brazilian public healthcare system (SUS), which covers the whole Brazilian population but of which approximately 22.5% are also covered by private insurance [13]. Data available in DATASUS are anonymized and encrypted and made available publicly. In addition, according to the Brazilian ethics Resolution No. 510 from April 7, 2016, studies of public domain information do not require approval from ethics committees [14].

### Population

**Fig 1:**
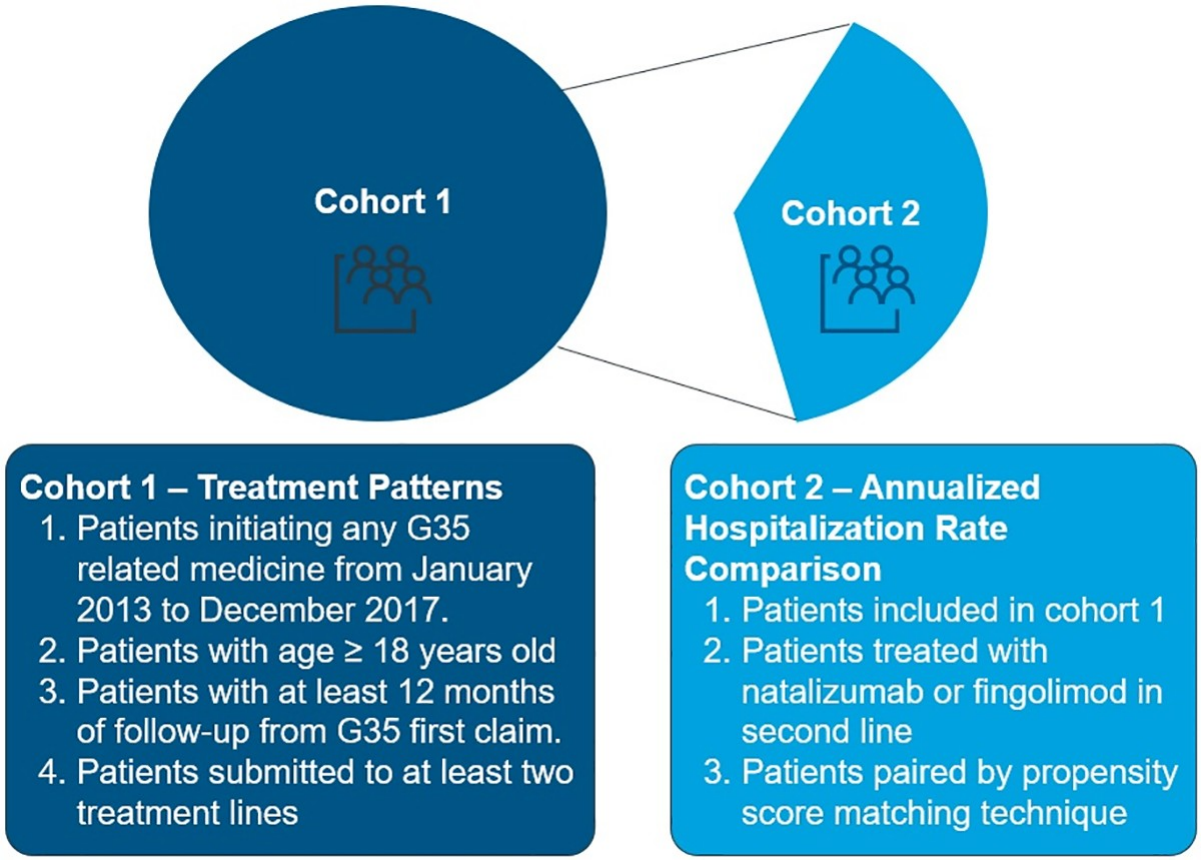
Key eligibility criteria for cohorts 1 and 2. G35, ICD10-G35 code for multiple sclerosis patients.

The present study included two distinct cohorts: cohort 1 to analyze RRMS treatment patterns and hospitalization profiles; cohort 2 to evaluate natalizumab and fingolimod AHR. Cohort 1 included all individuals who had at least one claim related to MS therapies, were aged ≥ 18 years old, had initiated MS treatment from January 2013 until December 2017, switched from first-line platform therapy, had at least 12 months of follow-up in the database and received at least two lines of treatments. In cohort 2, all patients from cohort 1 who were treated with fingolimod or natalizumab in second-line, switching from platform therapies, were included in the unmatched cohort. For analyses adjusted for cofactors, we applied two different approaches: a matched cohort for each treatment paired by a propensity score (PS) matching strategy (Fig 1); and inverse probability weight (IPW) in all cohort 2 patients. For both adjusted analyses, we considered the following variables: time between first ICD-10 G35 report and initiation of first-line therapy, sex, age, duration of first-line therapy and region of the country. Only patients starting treatment from 2013 were included. Patients with claims in 2013 who started treatment before 2013 were excluded.

### Information systems

DATASUS is an administrative database that contains information about procedures performed in SUS and includes the whole Brazilian population (209 million), of which more than 160 million are exclusively dependent on SUS. The administrative claims data are presented as procedure codes from billing records and include demographic information, clinical information in the patient profile, number of procedures, costs and other information. The information reported includes both diagnosis and therapeutic procedures. All outpatient and inpatient procedures available nationally in SUS and reported in DATASUS (approximately 6,500 procedures) are included in the *Sistema de Gerenciamento da Tabela de Procedimentos*, *Medicamentos e OPM do SUS* (SIGTAP) procedure list.

Data on inpatient and outpatient procedures are thus downloadable from DATASUS website, from which we collected all the information used in this study. The outpatient and inpatient databases, however, are not linked by a unique patient identifier; therefore, a probabilistic record linkage was performed to achieve longitudinal patient data in both settings. On the other hand, low-complexity drugs (e.g., statins, hypoglycemiants, antihypertensives) are not reported in DATASUS, and some low-complexity outpatient procedures (e.g., physician visits, blood tests) are reported in an aggregated form, not individually for each patient.

### Data extraction and assessments

#### Data extraction

After we identified all treated MS patients, we used a probabilistic linkage approach to obtain longitudinal patient data from both inpatient and outpatient settings. The probabilistic record linkage was performed using information such as date of birth, ZIP code and ICD-10 diagnosis code [15]. Patients entered the cohort only once, at the first study entry date when all inclusion and exclusion criteria were met. The data source was cleaned to exclude all individuals with inconsistent data (e.g., excessive missing data). For this study, inpatient procedures with more than one day of duration were considered hospitalization to avoid bias with patients who were hospitalized due to treatment requirements, such as intravenous infusion for natalizumab and monitoring for fingolimod.

#### Statistical analysis

To compare fingolimod and natalizumab treatments in second-line treatment and to adjust for cofactor bias due to the heterogeneity of the sample, we performed two strategies in the cohort 2 analysis: one-to-one PS matching and IPW to address existing differences at the baseline. PS were calculated by logistic regression analysis, using 1:1 nearest-neighbor matching [16], with a maximum difference among the PS of 0.1. In the IPW analysis, the weight corresponded to the inverse of the PS [17]. Covariates for the adjustment strategies included age at first claim, sex, region, time from first MS claim to the beginning of the first treatment, drug in first-line treatment and time in first-line treatment. After adjustments, we compared first-line treatment AHR and reduction of AHR among matched cohorts of patients treated with fingolimod or natalizumab in second-line treatment.

Demographics, treatment patterns and switch patterns were analyzed for cohort 1, while comparison of AHR between natalizumab and fingolimod were conducted for cohort 2. Demographic data, treatment patterns, switching patterns, outpatient and inpatient procedures and healthcare resource consumption were analyzed. Data are reported as continuous variables (quantitative ones) and were summarized by mean, standard deviation (SD), median and minimum and maximum. For the comparison of AHR between the groups, a Poisson test was applied for the adjusted analysis. P<0.05 was used to determine statistical significance. The statistical program used was R software version 3.5.0.

## Results

Of 29,410 patients with MS treated in the specified period, a total of 2,876 patients were included in cohort 1 for these analyses. The majority of eligible patients in cohort 1 were female (73.2%) with a median age of 33 years (Table 1).

**Table 1:** Demographics and characteristics of patients with MS.

Characteristic	Number of Patients (N = 2,876)
Age at first MS claim, years	
Mean (SD)	34.7 (10.5)
Median (range)	33 (18–76)
Sex, n (%)	
Female	2,105 (73.2)
Ethnicity, n (%)	
White	911 (31.7)
Black	47 (1.7)
Mixed race	349 (12.1)
Yellow	24 (0.8)
Unknown	1,545 (53.7)

MS, multiple sclerosis; SD, standard deviation.

In second-line treatment, 540 patients from cohort 1 were treated with natalizumab and 465 with fingolimod. When evaluated by PS strategy, a total of 375 patients were matched on each treatment. In the IPW analysis, no patients were excluded by the matching strategy (Table 2).

**Table 2:** Comparison of patient characteristics for unmatched cohort, propensity score matched cohort and inverse probability weighting adjusted cohort.

** **	**Unmatched Cohort**			**Propensity Score Matched Cohort**			**Inverse Probability Weighting**		
	Fingolimod (n = 465)	Natalizumab (n = 540)	P value*	Fingolimod (n = 375)	Natalizumab (n = 375)	P value*	Fingolimod (n = 465)	Natalizumab (n = 540)	P value*
Age at first MS claim, mean (SD), *years*	34.2 (10.0)	34.6 (10.4)	0.56	34.04 (9.9)	34.6 (10.4)	0.46	34.5 (10.0)	34.6 (10.3)	0.96
Female, *n* (%)	331 (71.2)	375 (69.4)	0.55	265 (70.7%)	262 (69.9)	0.81	327 (70.3)	382 (70.5)	0.99
Region of the country, *n* (%)									
Centre-West	52 (11.2)	39 (7.2)		38 (10.1)	37 (9.9)		43 (9.2)	49 (9.1)	
Northeast	52 (11.2)	85 (15.7)		47 (12.5)	50 (13.3)		61 (13.2)	72 (13.3)	
North	3 (0.7)	9 (1.7)	**<0.0001**	3 (0.8)	4 (1.1)	0.96	6 (1.3)	7 (1.2)	0.99
Southeast	257 (55.3)	365 (67.6)		239 (63.7)	242 (64.5)		288 (62.1)	336 (62.0)	
South	101 (21.7)	42 (7.8)		48 (12.8)	42 (11.2)		66 (14.3)	79 (14.5)	
Time from first claim to treatment start in months (SD)	21.4 (114.9)	24.5 (121.1)	0.68	19.8 (103.4)	29.9 (141.7)	0.27	30.3 (146.7)	26.3 (126.5)	0.64
Time in first-line treatment in years (SD)	1.8 (1.2)	1.4 (1.1)	**<0.0001**	1.6 (1.0)	1.6 (1.2)	0.92	1.6 (1.1)	1.6 (1.2)	0.93
First-line drug (%)									
Interferon β-1a IM	131 (28.2)	139 (25.7)		102 (27.2)	103 (27.5)		126 (27.2)	147 (27.2)	
Interferon β-1b SC	60 (12.9)	84 (15.6)		54 (14.4)	47 (12.5)		65 (14.0)	79 (14.6)	
Glatiramer	154 (33.1)	160 (29.6)	0.29	118 (31.5)	116 (30.9)	0.85	146 (31.4)	168 (31.0)	0.99
Interferon β-1a SC	120 (25.8)	157 (29.1)		101 (26.9)	109 (29.1)		127 (27.4)	148 (27.3)	

* Student's t-test

### Treatment patterns analysis

**Fig 2:**
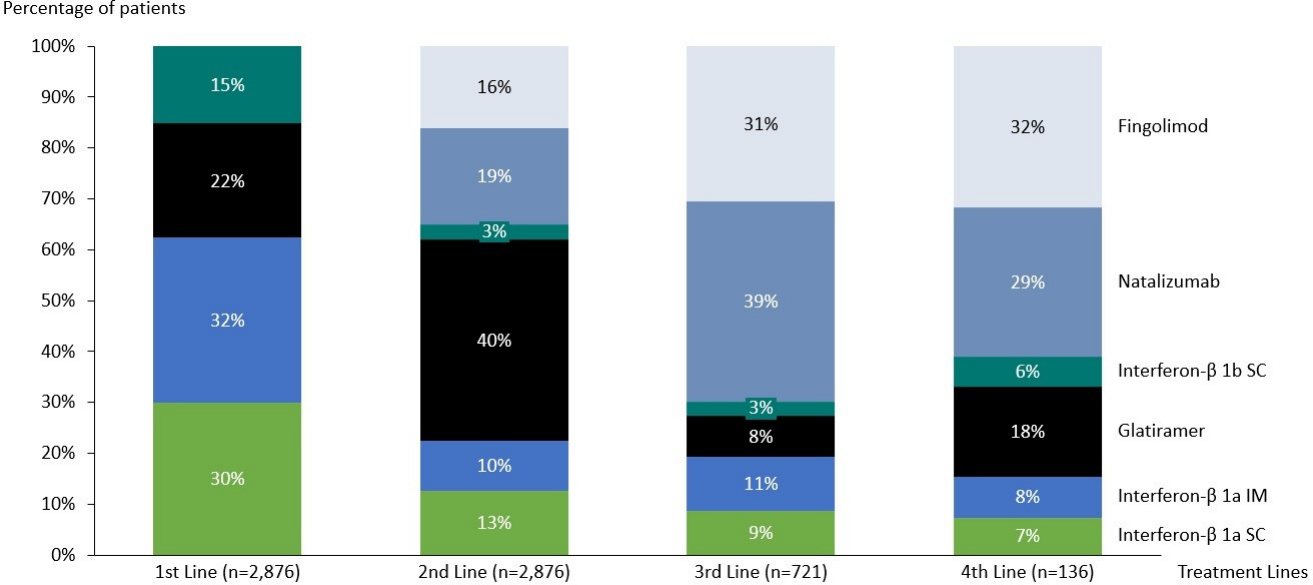
Treatment patterns in first to fourth line among the included patients.

For the treatment pattern analysis, all patients from cohort 1 (n = 2,876) were included. According to the inclusion criteria, all cohort 1 patients had received a second-line therapy. The proportion of patients who received third-, fourth- and further lines of treatment was 25.1%, 6.4% and 1.6%, respectively. Fig 2 summarizes the treatment regimens administered to the MS patients in first- to fourth-line treatments and the number of patients who received each treatment.

As second-line therapy, glatiramer was the most frequently used drug (n = 1,137, 39.6%), followed by natalizumab (n = 540, 18.8%) and fingolimod (n = 465, 16.2%). Natalizumab and fingolimod had higher median (range) treatment durations in second-line therapy, 16.02 months (0–66.09) and 14.01 months (0.95–33.09), respectively, followed by interferon β-1a IM (13.06 months [0–66.09]).

The most commonly used third-line therapies were natalizumab (39.4%), fingolimod (30.5%) and interferon β-1a IM (10.5%), with fingolimod having the highest median treatment duration (12.01 months [0.99–32.11]), followed by natalizumab (11.04 months [0.99–51.05]). As fourth-line therapy, interferon β-1a IM and natalizumab had higher median treatment durations (9.01 months [0.99–40] for each).

### Treatment switching analysis

All 2,876 patients included in the cohort 1 analysis received platform therapies in first-line treatment. The most common switches in first-line treatment were from interferon β-1a IM to glatiramer (52.09%), and the same percentage switched from interferon β-1a SC to glatiramer. Of the 1,140 patients who received glatiramer in second-line treatment, 17.01% switched to natalizumab and 10.52% to fingolimod in third-line treatment. Of the 437 patients who used interferon β-1b SC in first-line treatment, 46.91% switched to glatiramer and 19.22% to natalizumab.

Considering the 646 patients who used glatiramer in first-line treatment, 28.63% used interferon β-1a SC and 24.76% switched to natalizumab in second-line treatment. Of the 540 patients who used natalizumab in second-line treatment, only 12.39% switched to a third-line treatment (see S1 File for treatment sequence starting in Interferon 1b SC; S2 File starting in Interferon 1a –SC; S3 File starting in Interferon 1a –IM; S4 File starting in glatiramer and S5 File with all possibilities).

### Annualized hospitalization rate

In the unmatched natalizumab cohort, patients in first-line treatment with platform therapies had an AHR of 0.124 (CI_95%_: 0.10–0.15) and on second-line treatment with natalizumab, an AHR of 0.038 (CI_95%_: 0.02–0.05), representing a rate difference of 0.086 (CI_95%_: 0.06–0.1, p<0.011). In the unmatched fingolimod cohort, patients in first-line treatment with platform therapies had an AHR of 0.097 (CI_95%_: 0.08–0.12), with an AHR of 0.061 (CI_95%_: 0.04–0.08) for second-line treatment with fingolimod, representing a rate difference of 0.035 (CI_95%_: 0.01- 0.06, p = 0.02) (Table 3).

**Table 3:** Annualized hospitalization rates of fingolimod and natalizumab in second-line treatment for unmatched cohort, propensity score matched cohort and inverse probability weighting cohort.

	Unmatched Cohort		Propensity Score Cohort (Matched Cohort)		Inverse Probability Weighting	
	AHR (CI_95%_)	P value*	AHR (CI_95%_)	P value*	AHR (CI_95%_)	P value*
***Annualized Hospitalization Rate***	***Natalizumab (n = 540)***		***Natalizumab (n = 375)***		***Natalizumab (n = 540)***	
Platform therapy in first-line treatment	0.124 (0.10–0.15)	-	0.100 (0.07–0.13)	-	0.120 (0.10–0.14)	-
Natalizumab in second-line treatment	0.038 (0.02–0.05)	-	0.036 (0.02–0.05)	-	0.036 (0.02–0.05)	-
*Difference between first- and second-line AHR*	0.086 (0.06–0.11)	**<0.01**	0.064 (0.03–0.09)	**<0.0001**	0.084 (0.06–0.11)	**<0.0001**
***Annualized Hospitalization Rate***	***Fingolimod (n = 465)***		***Fingolimod (n = 375)***		***Fingolimod (n = 465)***	
Platform therapy in first-line treatment	0.097 (0.08–0.12)	-	0.089 (0.07–0.11)	-	0.101 (0.08–0.12)	-
Fingolimod in second-line treatment	0.061 (0.04–0.08)	-	0.067 (0.04–0.09)	-	0.079 (0.06–0.10)	-
*Difference among first- and second-line AHR*	0.035 (0.01–0.06)	**0.02**	0.022 (-0.02–0.05)	0.20	0.023 (-0.01–0.06)	0.17
***Difference in second-line AHR–fingolimod vs*. *natalizumab***	N/A		0.032 (0.003–0.06)	**0.03**	0.043 (0.02–0.07)	**0.002**

* Poisson test

When evaluating patients from the PS matched cohort 2 (Table 2), characteristics between groups were balanced, with 375 paired patients in each group. Patients treated with natalizumab in second-line treatment had a 0.064 (CI_95%_: 0.03–0.09, p<0.0001) difference rate compared with first-line treatment, with a reduction of 64.1%, while patients treated with fingolimod had a numerical but not statistically significant AHR reduction compared with first-line treatment (0.022 [CI_95%_: -0.02–0.05], p = 0.20), representing a reduction of 24.2% (Table 3). Additionally, the comparison of AHR with second-line treatments showed a higher reduction with natalizumab (natalizumab: 0.064 vs fingolimod 0.022; p = 0.03).

In the IPW adjusted analysis of cohort 1, characteristics among groups were also balanced (Table 2). In second-line treatment, patients treated with natalizumab had an 0.084 difference (CI_95%_: 0.06–0.11, p<0.0001) in AHR compared with first-line treatment, with a reduction of 70.0%, while patients treated with fingolimod had a numerical but not statistically significant AHR reduction compared with first-line (0.023 [CI_95%_: -0.01–0.06], p = 0.17, representing a reduction of 22.3%. As observed in the PS analysis, the comparison of reduction in AHR between second-line treatments also presented a statistical significantly difference in the IPW analysis (natalizumab 0.084 vs fingolimod 0.023; p = 0.002) (Table 3).

### Hospitalization profile

Treatment of MS relapse was the most frequent procedure in the inpatient setting, accounting for 75.99% of all procedures, with a mean of 24.47 records (SD: 27.05) and median of 16 records (min-max: 0–179) per hospitalized patient (Table 4). Treatment of central motor neuron disease with/without amyotrophy was the second most frequent procedure (10.2%) with a mean of 3.28 records (SD: 10.47) per hospitalized patient (Table 4). During second-line treatment, the median total days of hospitalization was 6 (min-max: 2–489) and the mean was 13.08 (SD: 45.71). The median number of procedures was 19 (min-max: 2–307) and the mean was 29.03 (SD: 36.12).

**Table 4:** Total number and percentage of times used by procedure and number of times procedure was performed per hospitalized patient from cohort 1.

Procedure*	Total number of times	Percentage	Number of times procedure was performed per hospitalized patient	
			Mean (SD)	Median (Min-Max)
MS relapse treatment	10352	75.99	24.47 (27.05)	16 (0–179)
Central motor neuron disease treatment with/without amyotrophy**	1389	10.20	3.28 (10.47)	0 (0–77)
Physiotherapy for motor disorders	397	2.91	0.94 (12.21)	0 (0–247)
Prolonged treatment due to neurological disease	357	2.62	0.84 (15.12)	0 (0–307)
Intensive care/follow-up of physical rehabilitation patient (20 visits/month)	194	1.42	0.46 (4.88)	0 (0–77)
Physiotherapy for neuro-kinetic-functional disorders without complication	174	1.28	0.41 (3.20)	0 (0–53)
Cranium MRI	152	1.12	0.36 (1.03)	0 (0–12)
Diagnosis and/or emergency care in medical clinical	120	0.88	0.28 (2.59)	0 (0–44)
Intensive care/follow-up of physical rehabilitation patient (15 visits/month)	117	0.86	0.28 (3.59)	0 (0–63)
Cervical MRI	87	0.64	0.21 (0.61)	0 (0–4)

* Procedures defined according SIGTAP table.

** Clinical treatment of respiratory or neurologic complication in patients with central neuron diseases. Applicable for the following diseases: Huntington disease, congenital ataxy, cerebellar ataxy, hereditary spastic paraplegy, other hereditary ataxies, spinal muscular athrophy, motor neuron disease and multiple sclerosis.

In a PS calculation comparing second-line treatment cohorts using natalizumab and fingolimod, fingolimod had a higher mean (54.4 [SD: 91.1]) and median 24 (min-max: 5–307) number of procedures compared with natalizumab (mean: 25.3 [SD: 21.1] and median: 20 [min-max: 2–76]) (Table 5). Patients treated with fingolimod also had longer duration in hospital with a mean of 56.7 days (SD: 152.09) compared with natalizumab with a mean of 10.53 days (SD: 7.28) (Table 5).

**Table 5:** Number of procedures during hospitalization and total days of hospitalization of fingolimod and natalizumab in second-line treatment for matched cohort 2.

Treatment Line	Therapy	Number of procedures during hospitalization		Total days–Hospitalized patients	
		Mean (SD)	Median (Min-Max)	Mean (SD)	Median (Min-Max)
2^nd^	Fingolimod	54.4 (91.1)	24 (5–307)	56.7 (152.09)	6.5 (4–489)
	Natalizumab	25.3 (21.1)	20 (2–76)	10.53 (7.28)	8 (2–27)

## Discussion

The most common MS treatments after failure of one platform treatment were glatiramer acetate, natalizumab and fingolimod, while the most common treatment sequence observed was interferon β-1a IM → glatiramer acetate → natalizumab. Natalizumab showed effectiveness in decreasing the AHR when used in second-line treatment after use of platform therapies, with an approximate reduction of 63% compared with first-line treatment. Fingolimod showed a statistically significant reduction in AHR only when analyzing the unmatched cohort.

Our data corroborate the findings of previously published studies, such as a longitudinal real-life study in Italy [18] that showed a greater reduction in the annualized relapse rate (ARR) in the natalizumab group than in the fingolimod group. Another study in Germany [19] demonstrated that natalizumab in second-line treatment reduced the ARR from 2.1 to 0.2, and a more recent study [20] suggests that switching to natalizumab is more effective in reducing relapse rate and short-term disability burden than switching to fingolimod. In fact, several studies suggest that natalizumab is superior to fingolimod in preventing MS relapses [9,10,12,20–24]. On the other hand, our data contrasts with other studies in which fingolimod and natalizumab had similar efficacy [25,26].

In addition, other similar studies also demonstrate that MS relapse represents an important economic burden to healthcare systems, with high treatment costs [27,28], and an important burden with respect to the quality of life and functional ability of patients [28,29].

Regarding the hospitalization profile, a high median number of times procedures were performed per hospitalized patient was observed for treatment of MS relapse. MS relapse treatment was the most frequent procedure for the MS patients observed during the study period, with an elevated number of times compared with other procedures performed per hospitalized patient. For second-line treatment, the mean duration of hospitalization was 13.08 days, similar to results from a Canadian study that reported a mean of 10.2 days [30]. An American [31] and a Spanish study [32] showed a lower number, with an average of 5 total days of hospitalization. Furthermore, in cohort 2, the mean duration of hospitalization for the matched cohort was higher for fingolimod than for natalizumab.

The strength of this study is the use of DATASUS, which covers most of Brazil´s population (77.5% of the population is exclusively dependent on SUS), thus providing detailed information on patients with multiple sclerosis. On the other hand, this study also has some limitations: this was a retrospective analysis from a database subject to data collection inconsistencies, and due to the nature of administrative claims data, limited clinical information is available, which could affect adjustments and outcome analysis. Because healthcare utilization claims data do not allow for detailed analysis of relapses, disease progression and improvement, some important clinical information, such as Expanded Disability Status Scale (EDSS) scores and existence of comorbidities, could not be included in the pairing methodology. In this context, the results of this study should be extrapolated carefully. Other limitations include the lack of reporting of low-complexity procedures in DATASUS.

## Conclusion

To the best of our knowledge, this is the first study to evaluate differences in treatment patterns for RRMS in SUS and to compare the AHR of natalizumab and fingolimod within the Brazilian healthcare system. This study provides real-world evidence of natalizumab’s effectiveness in terms of hospitalization rates compared with fingolimod, with decreased hospitalization rates in natalizumab-treated patients.

## Supporting information

S1 File Treatment sequence after Interferon 1b –SC in first line. (HTML)

S2 File Treatment sequence after Interferon 1a –SC in first line. (HTML)

S3 File Treatment sequence after Interferon 1a –IM in first line. (HTML)

S4 File Treatment sequence after Glatiramer in first line. (HTML)

S5 File Full treatment sequence including all first line treatment in the included population. (HTML)
